# Multivariate Time Series Change-Point Detection with a Novel Pearson-like Scaled Bregman Divergence

**DOI:** 10.3390/stats7020028

**Published:** 2024-05-13

**Authors:** Tong Si, Yunge Wang, Lingling Zhang, Evan Richmond, Tae-Hyuk Ahn, Haijun Gong

**Affiliations:** 1 Department of Mathematics and Statistics, Saint Louis University, St. Louis, MO 63103, USA; 2 Department of Mathematics and Statistics, University at Albany SUNY, Albany, NY 12222, USA; 3 Department of Computer Science, Saint Louis University, St. Louis, MO 63103, USA

**Keywords:** change-point detection, time-series data analysis, density ratio estimation, scaled Bregman divergence, random sampling

## Abstract

Change-point detection is a challenging problem that has a number of applications across various real-world domains. The primary objective of CPD is to identify specific time points where the underlying system undergoes transitions between different states, each characterized by its distinct data distribution. Precise identification of change points in time series omics data can provide insights into the dynamic and temporal characteristics inherent to complex biological systems. Many change-point detection methods have traditionally focused on the direct estimation of data distributions. However, these approaches become unrealistic in high-dimensional data analysis. Density ratio methods have emerged as promising approaches for change-point detection since estimating density ratios is easier than directly estimating individual densities. Nevertheless, the divergence measures used in these methods may suffer from numerical instability during computation. Additionally, the most popular α-relative Pearson divergence cannot measure the dissimilarity between two distributions of data but a mixture of distributions. To overcome the limitations of existing density ratio-based methods, we propose a novel approach called the Pearson-like scaled-Bregman divergence-based (PLsBD) density ratio estimation method for change-point detection. Our theoretical studies derive an analytical expression for the Pearson-like scaled Bregman divergence using a mixture measure. We integrate the PLsBD with a kernel regression model and apply a random sampling strategy to identify change points in both synthetic data and real-world high-dimensional genomics data of Drosophila. Our PLsBD method demonstrates superior performance compared to many other change-point detection methods.

## 1. Introduction

Change-point detection (CPD) has a number of applications across various real-world domains [1–5], for example, network analysis [6], motion detection and sensing [7], abnormal signal processing [8], the identification of differentially expressed genes [9,10], the inference of time-varying regulatory networks [11], and the evolutionary analysis of infectious disease with pandemic potential [12]. The primary objective of change-point detection is to identify times or intervals associated with abrupt transitions or shifts within various types of time series data, including categorical data [2], censored panel data [4], and pandemic data [12]. The identification of change points enhances our comprehension of system behaviors, such as understanding how the regulatory networks reconfigure at different stages of cell cycle progression. Change points split time series data into distinct segments or stages, each characterized by its statistical properties [13]. These rapid changes in properties may include changes in means, variances, or patterns within each segment.Statistical analysis considers a point in time to be a change point if there is a noticeable change in the distribution of observed data before and after that point.

Identifying change points in multivariate time-series datasets presents a significant challenge, especially when the number of variables far exceeds the number of observations [14], as often encountered in time series of omics data. Over the past decades, numerous methods, including supervised [15–17] and unsupervised [18,19], parametric [20] and non-parametric [13,21–23] change-point detection techniques have been developed. Most of these methods utilize the divergence between two probability distributions of observations to identify significant changes in the data. If the divergence between two distributions is notably large, the time point is considered a change-point candidate. Most traditional parametric approaches require distributional assumptions to detect changes in distribution parameters, such as hierarchical models [5], cumulative sum (CUSUM) methods [24,25] and Shiryaev–Roberts methods [26]. The CUSUM method is one of the most popular change-point algorithms which estimates the cumulative log-likelihood ratio sequence to identify change points. A change point exists when the cumulative sum exceeds a specified threshold, so the CUSUM has served as the foundation for numerous CPD approaches. For instance, the INSPECT algorithm [27] estimates change points by conducting a sparse singular value decomposition on the CUSUM transformation matrix and identifying optimal projection directions corresponding to structure changes between consecutive stationary stages. Our previous studies [28] applied the INSPECT algorithm to study the time-varying regulatory networks. The Shiryaev–Roberts method is similar to the CUSUM method but it replaces the log-likelihood ratio in the CUSUM statistics with the likelihood ratio. Both CUSUM and Shiryaev–Roberts methods require complete knowledge of distributions before and after the change points to learn the likelihood information. To circumvent distributional assumptions, some non-parametric and generative approaches have been proposed, for example, kernel maximum mean discrepancy (MMD) [29] for two-sample tests, maximum kernel Fisher discriminant ratio-based method [30] and nearest neighbors method [31]. Sun et al.’s work [32] proposed a neural stochastic differential equations (SDEs) method to learn the change points under the framework of generative adversarial networks (GANs). These non-parametric and generative methods can provide more flexibility in change-point detection compared to parametric methods because there is no parameter assumption.

The applications of change-point detection can be divided into online (real-time) detection and offline (retrospective) detection. Numerous packages, such as Finder [33], NEWMA [34], and onlineBcp [35], have been developed for the online and offline change-point detection tasks. We focus on the offline change-point detection, which detects change points when all the observations have been collected. Many classical change-point detection methods, for example, Bayesian change detection [36], require the estimation of individual distributions before and after change points. However, the direct estimation of data distributions in high-dimensional data is often unrealistic in real-world scenarios, especially genomics data with thousands of genes. To overcome this limitation, various density ratio estimation methods have been developed to directly estimate the density ratios instead of estimating individual densities, including the kernel mean matching [37], ratio matching [38], probabilistic classification approaches [39], adversarial density ratio estimation [40], Kullback–Leibler (KL) importance estimation procedure (KLIEP) [41], unconstrained least-squares importance fitting (uLSIF) [42], and relative uLSIF (RuLSIF) [43] approaches.

Several papers, including Liu et al. [44] and Aminikhanghahi et al. [45], have provided comprehensive surveys of various change-point detection methods and compared their performance. These methods utilize various divergence measures such as the Kullback–Leibler (KL), Pearson, and α-relative Pearson divergence to quantify the dissimilarity between data distributions and detect the change points. However, these divergence functions do not satisfy the properties of symmetry and triangle inequalities, making them non-metric measures. Additionally, some of these divergence functions may introduce severe unboundedness issues. The density ratio estimation methods have demonstrated strong performance in previous change-point detection studies. Among these methods, the RuLSIF approach outperforms many other approaches; however, the α-relative Pearson divergence used in the RuLSIF approach does not measure the dissimilarity between two distributions of data but a mixture of distributions.

The rest of the paper is outlined as follows: In the Materials and Methods section, we provide a brief overview of existing density ratio estimation-based algorithms, highlighting their key features and limitations. We then propose a new approach called a Pearson-like scaled Bregman divergence-based (PLsBD) density ratio estimation algorithm and a random sampling approach for change-point detection. This method is designed to overcome the limitations encountered in traditional density ratio-based approaches. Compared with existing density ratio estimation methods, the major novelty of the PLsBD lies in its ability to measure the disparity between two individual distributions of data, rather than a mixture of distributions, without introducing the unboundedness issues. In the Results section, the proposed methods are applied to identify change points using both synthetic data and high-dimensional real-world omics data. Lastly, we discuss the advantages and disadvantages of the proposed technique and potential directions for future research and development.

## 2. Materials and Methods

In statistics, a change point signifies a transition between two different states in a stochastic process, where each state is characterized by a distinct probability distribution. The goal of change-point detection is to learn the underlying probability distributions corresponding to the time series data both before and after the change-point. Let $ϒ(t)\in {\mathbb{R}}^{d}$ be a sequence of d-dimensional time series data. To identify the change points, a divergence function is usually used to measure the dissimilarity between two segments of timeseries data, which follow different probability distributions P(ϒ) and Q(ϒ), with density functions p(y) and q(y), respectively. There are two main approaches to learn the density functions. The direct density estimation methods estimate the density functions p(y) and q(y) individually from sampled data. By comparing these estimated density functions, the changes in densities can be detected, leading to the identification of change points. In contrast, density ratio estimation methods focus on the direct estimation of the density ratio $\frac{p(y)}{q(y)}$ without the requirement to individually estimate each density function. Changes in the density ratio can detect the transitions in the underlying data distributions, thereby identifying change points.

### 2.1. Density Ratio Estimation Methods

The Kullback–Leibler (KL) divergence and Pearson (PE) divergence, which belong to the f-divergence (or Ali–Silvey–Csiszar divergence) family, have been widely used to measure the dissimilarity between two distributions P and Q with densities p and q, respectively:

(1) $$D_{KL}(p\|q)=\int p(y)\log\left(\frac{p(y)}{q(y)}\right)dy,$$

(2) $$D_{PE}(p\|q)=\frac{1}{2}\int q(y){\left(\frac{p(y)}{q(y)}-1\right)}^{2}dy.$$

The Kullback–Leibler importance estimation procedure (KLIEP) [41] and unconstrained least-squares importance fitting (uLSIF) [42] are two widely adopted change-point detection methods that utilize the KL and Pearson divergence, respectively, for estimating density ratios. Since the optimization problems for both KLIEP and uLSIF are convex, a unique global optimal solution always exists, which ensures the stability and reliability of the estimation process. The optimal values of some parameters can be effectively estimated using cross-validation methods. While the KLIEP is computationally expensive due to the logarithmic functions and is sensitive to outliers, the uLSIF method offers more computational efficiency and robustness compared to the KLIEP. However, a significant weakness shared by both methods is the potential for the density ratio p/q to become unbounded under certain conditions when employing the KL and Pearson divergences. This unboundedness can lead to challenges in practical applications and influence the stability and effectiveness of the methods.

To address the potential issue of unboundedness in the density ratio function within the KLIEP and uLSIF method, a relative uLSIF (RuLSIF) approach [43] was introduced to replace the Pearson divergence with an α-relative Pearson divergence:

(3) $$D_{\alpha PE}\left(p\|q_{\alpha }\right)=\frac{1}{2}\int q_{\alpha }(y){\left(\frac{p(y)}{q_{\alpha }(y)}-1\right)}^{2}\text{d}y,$$

where $q_{\alpha }(y)=\alpha p(y)+(1-\alpha )q(y)$, and 0≤α≤1. The relative ratio $\frac{p(y)}{q_{\alpha }(y)}$ has an upper bound 1/α, and the estimation procedure closely resembles that of the uLSIF method.

The divergence functions utilized in the KLIEP, uLSIF, and RuLSIF methods, such as KL, Pearson, and α-relative Pearson divergence, do not meet all the criteria for metric measures. A divergence function D(p‖q) is classified as a metric if it satisfies the following conditions:

- Non-negativity: D(p‖q)≥0 with equality if and only if p=q;
- Symmetry: D(p‖q)=D(q‖p);
- Triangle inequality: D(p‖q)≤D(p‖r)+D(r‖q) for all probability distributions p, q, r.

These divergence functions violate the properties of symmetry and the triangle inequality. Though the RuLSIF-based approach outperforms many other approaches, another significant drawback of the α-relative Pearson divergence used in the RuLSIF method is that it measures dissimilarity between p and a mixture distribution $q_{\alpha }$ rather than between two individual distributions. Consequently, the RuLSIF method is sensitive to the value of α. Our studies using RuLSIF method found that a large α value could introduce numerical instability in computations. To overcome the limitations of existing density ratiobased methods, especially the RuLSIF method, we introduce a novel approach termed the Pearson-like scaled Bregman divergence-based (PLsBD) density ratio estimation method for change-point detection. The PLsBD can measure the disparity between two individual distributions of two subsequences of data, rather than a mixture of distributions. Next, we present a comprehensive theoretical analysis of the PLsBD method.

### 2.2. Pearson-like Scaled Bregman Divergence (PLsBD)

Given two measures P and Q on 𝒴 with densities p and q, respectively, the separable Bregman divergence [46] is defined as

$$D_{BR}(p\|q)=\int_{y}f(p(y))-f(q(y))-f^{\prime }(q(y))(p(y)-q(y))dy,$$

where $f:{\mathbb{R}}^{+}\to \mathbb{R}$ is a convex function, and $f^{\prime }$ is its right derivative.

Introducing a third measure M with density m leads to the definition of a scaled Bregman divergence [47], which encompasses a broad family of divergences. This divergence is expressed as:

(4) $$D_{sBR}(p\|q;m)=\int_{𝒴}\left[f\left(\frac{p(y)}{m(y)}\right)-f\left(\frac{q(y)}{m(y)}\right)-f^{\prime }\left(\frac{q(y)}{m(y)}\right)\left(\frac{p(y)}{m(y)}-\frac{q(y)}{m(y)}\right)\right]m(y)dy,$$

where dM=m(y)dy. Different choices of the measure M and function f yield various well-known divergence functions. For instance, if m(y)=q(y) and assuming f(1)=0, we obtain the f-divergence

$$D_{f}(p\|q)=\int_{𝒴}q(x)f\left(\frac{p(y)}{q(y)}\right)dy.$$

When f(t)=t log t, the scaled Bregman divergence reduces to the KL divergence, which does not rely on the measure M. Alternatively, when $f(t)=\frac{1}{2}{\left(t-1\right)}^{2}$, we derive a new divergence termed the Pearson-like scaled Bregman divergence (PLsBD). This divergence bears partial resemblance to the Pearson divergence and is defined as:

(5) $$D_{PL}(p\|q;m)=\frac{1}{2}\int_{𝒴}m(y){\left(\frac{p(y)}{m(y)}-\frac{q(y)}{m(y)}\right)}^{2}dy.$$

If the measure $M^{\prime }\text{s}$ density m(y)=q(y), Equation (5) is reduced to the Pearson divergence used in the uLSIF algorithm, so the Pearson divergence is a special instance of the Pearson-like divergence. If $m(y)=q_{\alpha }(y)$, it is evident that the Pearson-like scaled Bregman divergence remains bounded, just like the α-relative Pearson divergence, which ensures the stability and robustness of the divergence measure in capturing the differences between the distributions p and q. One significant advantage of the Pearson-like scaled Bregman divergence is that given a measure $m,D_{PL}(p\|q;m)$ quantifies the discrepancy between two individual distributions p and q, so it could potentially serve as a metric measure under certain conditions. We established the following results:

#### Theorem 1.

*Given a measure* m, *the square root of the Pearson-like scaled Bregman divergence between two distributions* p *and* q, $\sqrt{D_{PL}(p\|q;m)}$, *forms a metric provided that the measure* m *is not dependent on* p *and* q.

##### Proof.

It is evident that $D_{PL}(p\|q;m)\geq 0$ and $D_{PL}(p\|p;m)=0$. Additionally, if m(y) is independent of p(y) and q(y), from Equation (5), the symmetry condition also holds: $D_{P_{L}}(p\|q;m)=D_{PL}(q\|p;m)$. Finally, applying Minkowski’s inequality, ${\left(\int_{ϒ}|f+g{|}^{p}d\mu \right)}^{1/p}\leq {\left(\int_{ϒ}|f{|}^{p}d\mu \right)}^{1/p}+{\left(\int_{ϒ}|g{|}^{p}d\mu \right)}^{1/p}$, when p=2, we can establish that the square root of the Pearson-like scaled Bregman divergence $\sqrt{D_{PL}(p\|q;m)}$ satisfies the triangle inequality. □

#### Lemma 1.

*If* M *is a uniform scale, then the Pearson-like scaled Bregman divergence* $D_{PL}(p\|p;m)$ *simplifies to* $L^{2}$ *distance, which is symmetric and satisfies the triangle inequality*.

Theorem 1 has demonstrated that if the density measure m is independent of p and q, $D_{P_{L}}(p|q;m)$ qualifies as a metric. Nevertheless, estimating the individual density functions remains necessary. The density ratio estimation method offers an advantage by estimating the density ratio $\frac{p(ϒ)}{q(ϒ)}$ instead of the individual density function. To accommodate this, we introduce a mixture density measure for m that depends on p and q:

(6) $$m_{\alpha }(y)=\alpha p(y)+(1-\alpha )q(y),\;\text{where}\;0\leq \alpha \leq 1.$$

#### Lemma 2.

*If* $m_{0}(y)=\frac{p(y)+q(y)}{2}$, *then*, $D_{PL}\left(p\|q;m_{0}\right)=D_{P_{L}}\left(q\|p;m_{0}\right)$.

It is known that the KL, Pearson, and α-relative Pearson divergences are not symmetric. Previous work had to construct a symmetric variant to estimate dissimilarity. Despite employing the mixture density measure $m_{\alpha }$, $D_{P_{L}}(p|q;m)$ does not always satisfy the symmetry and triangle inequality properties. However, Lemma 2 establishes that by selecting a specific measure $m_{0}$ when α=1/2, we can obtain a symmetric Pearson-like scaled Bregman divergence. Consequently, the *Pearson-like* scaled Bregman divergence is a good measure to quantify distribution differences, capable of mitigating the limitations of the KLIEP, uLSIF, and RuLSIF algorithms, which rely on the KL divergence, Pearson divergence, and α-relative Pearson divergence. Furthermore, we derived the following theoretical result based on different values of α, which can be used for change-point detection.

#### Theorem 2.

*Given the measure* $m_{\alpha }(y)=\alpha p(y)+(1-\alpha )q(y)$, *the Pearson-like scaled Bregman divergence can be expressed as* $D_{PL}\left(p\|q;m_{\alpha }\right)=\frac{1}{2}E_{p}\left(r_{\alpha }(ϒ)\right)-\frac{2-\alpha }{2(1-\alpha )}E_{q}\left(r_{\alpha }(ϒ)\right)+\frac{1}{2(1-\alpha )}$, *where* $r_{\alpha }(ϒ)=\frac{p(ϒ)}{m_{\alpha }(ϒ)}$, *and* $E_{p}$ *and* $E_{q}$ *denote the calculation of means with respect to the densities* p *and* q, *respectively*.

##### Proof.

The Pearson-like Bregman divergence, constructed based on the mixture measure $m_{\alpha }(y)$, is contingent upon the relative density ratio $r_{\alpha }(ϒ)=\frac{p(ϒ)}{m_{\alpha }(ϒ)}$, using the equation $m_{\alpha }(y)=\alpha p(y)+(1-\alpha )q(y),D_{PL}\left(p\|q;m_{\alpha }\right)$ can be decomposed as

$$D_{PL}\left(p\|q;m_{\alpha }\right)=\frac{1}{2}\int_{𝒴}m_{\alpha }(y){\left(\frac{p(y)}{m_{\alpha }(y)}-\frac{q(y)}{m_{\alpha }(y)}\right)}^{2}dy\;\;\;\;\;=\frac{1}{2}\int_{𝒴}\left(p(y)r_{\alpha }(y)-2q(y)r_{\alpha }(y)+q(y)\frac{1-\alpha r_{\alpha }(y)}{1-\alpha }\right)dy\;\;\;\;\;=\frac{1}{2}E_{p}\left(r_{\alpha }(Y)\right)-\frac{2-\alpha }{2(1-\alpha )}E_{q}\left(r_{\alpha }(Y)\right)+\frac{1}{2(1-\alpha )}.$$

□

The estimation of the Pearson-like scaled Bregman divergence does not require an individual estimation of densities p and q. Using the relative density ratio estimator ${\hat{r}}_{\alpha }(ϒ)$, the aforementioned PLsBD can be approximated by computing the empirical average:

(7) $${\hat{D}}_{PL}\left(p\|q;m_{\alpha }\right)\approx \frac{1}{2n_{p}}\sum_{i=1}^{n_{p}}\left({\hat{r}}_{\alpha }\left({ϒ}_{i}^{p}\right)\right)-\frac{2-\alpha }{2(1-\alpha )n_{q}}\sum_{i=1}^{n_{q}}\left({\hat{r}}_{\alpha }\left({ϒ}_{i}^{q}\right)\right)+\frac{1}{2(1-\alpha )},$$

where the superscript p and q indicate that ${ϒ}_{i}$ is sampled from the distributions p(y) and q(y), respectively.

#### Lemma 3.

$D_{PL}\left(p\|q;m_{\alpha }\right)=D_{\alpha PE}\left(p\|q_{\alpha }\right)+\frac{\left(2-\alpha \right)}{2(1-\alpha )}\left(1-E_{q}\left(r_{\alpha }(ϒ)\right)\right)\geq D_{\alpha PE}\left(p\|q_{\alpha }\right)+\frac{\left(\alpha -2\right)}{2\alpha }$. *That is, the* α-*relative Pearson divergence, augmented by* $\frac{\left(\alpha -1\right)}{2\alpha }$, *serves as a lower bound for the Pearson-like scaled Bregman divergence*.

##### Proof.

Using the result of the α-relative Pearson divergence from Liu et al.’s work [44], it is easy to obtain $D_{PL}\left(p\|q;m_{\alpha }\right)=D_{\alpha PE}\left(p\|q_{\alpha }\right)+\frac{\left(2-\alpha \right)}{2(1-\alpha )}\left(1-E_{q}\left(r_{\alpha }(ϒ)\right)\right)$. Since $r_{\alpha }(ϒ))\leq \frac{1}{\alpha }$, we have $D_{PL}\left(p\|q;m_{\alpha }\right)\geq D_{\alpha PE}\left(p\|q_{\alpha }\right)+\frac{\left(\alpha -2\right)}{2\alpha }$. □

Previous studies [23,41,42] have demonstrated that the discrepancy computed by both Kullback–Leibler divergence and Hellinger divergence is bounded by the discrepancy calculated by the Pearson divergence. Lemma 3 elucidates that the proposed Pearson-like scaled Bregman divergence exhibits greater sensitivity to changes in time series data compared to the α-relative Pearson divergence, and this finding was further supported by a subsequent data analysis.

Our theoretical studies have demonstrated that the PLsBD behaves as a metric if the third measure m is independent on p and q. Introducing a mixture measure $m_{\alpha }$ to estimate density ratios may lead to situations where the PLsBD does not always satisfy the triangle inequality. Nevertheless, compared to other density ratio estimation methods, the PLsBD method provides notable advantages and innovations in quantifying distribution disparity. It not only addresses the issue of unboundedness but also evaluates dissimilarity between two individual data distributions rather than a mixture distribution in the RuLSIF method.

### 2.3. Kernel-Based Density Ratio Estimation

The density ratio $r_{\alpha }(ϒ)=\frac{p(ϒ)}{m_{\alpha }(ϒ)}$ can be estimated using a Gaussian kernel regression method similar to Liu et al.’s work [44], which is modeled by

(8) $$r_{\alpha }(ϒ;\theta )=\sum_{l=1}^{n}\theta_{l}K\left(ϒ,{ϒ}_{l}\right),$$

where $\theta ={\left(\theta_{1},\ldots ,\theta_{n}\right)}^{⊤}$ are parameters learned from the data, and $K\left(ϒ,{ϒ}_{l}\right)$ is a Gaussian kernel expressed as:

$$K\left(ϒ,{ϒ}_{l}\right)=\exp\left(-\frac{{\left\|ϒ-{ϒ}_{l}\right\|}^{2}}{2\sigma^{2}}\right),$$

with a parameter σ(>0) which can be estimated using a cross-validation method.

The optimal values of the parameters θ can be learned by minimizing either the absolute error loss $\left(L_{1}\right)$ or squared error loss $\left(L_{2}\right)$, which quantifies the difference between $r_{\alpha }(ϒ)\;\text{and}\;r_{\alpha }(ϒ;\theta )$. Given the density measure $m_{\alpha }$, the $L_{1}$ loss is written as

$$J_{L_{1}}(\theta )=\int_{𝒴}\left|r_{\alpha }(ϒ)-r_{\alpha }(ϒ;\theta )\right|m_{\alpha }(ϒ)dϒ.$$

The $L_{2}$ squared error loss, given the density measure $m_{\alpha }$, can be expressed as

(9) $$J_{L_{2}}(\theta )=\frac{1}{2}\int_{𝒴}{\left(r_{\alpha }(ϒ)-r(ϒ;\theta )\right)}^{2}m_{\alpha }(ϒ)dϒ.$$

This work minimizes the squared error loss given in Equation (9) to estimate the model parameters for change-point detection. The preference to using the squared error $L_{2}$ loss over the absolute error $L_{1}$ loss in the change-point detection is apparent when considering the observations around the change points. The $L_{2}$ loss yields notably larger values for observations near the change points compared to the $L_{1}$ loss, which makes it more sensitive to deviations from the expected behavior around change points. Furthermore, the differentiability of the squared error loss facilitates its implementation in the optimization algorithm.

Similar to the RuLSIF method [44], if an ${𝓁}_{2}$ regularization term is included, minimizing the squared error loss in Equation (9) is equivalent to solving the following convex optimization problem:

(10) $$\min_{\theta \in {\mathbb{R}}^{n}}\left[\frac{1}{2}\theta^{⊤}\hat{H}\theta -{\hat{h}}_{p}^{⊤}\theta +\frac{\lambda }{2}\theta^{⊤}\theta \right],$$

where $\hat{H}$ is an n×n matrix with the $\left(𝓁,{𝓁}^{\prime }\right)$ th element the same as the results in [44]:

(11) $${\hat{H}}_{𝓁,{𝓁}^{\prime }}=\frac{\alpha }{n_{p}}\sum_{i=1}^{n_{p}}K_{il}^{p}K_{il^{\prime }}^{p}+\frac{\left(1-\alpha \right)}{n_{q}}\sum_{j=1}^{n_{q}}K_{jl}^{q}K_{jl^{\prime }}^{q},$$

and $K_{il}^{p/q}=K\left({ϒ}_{i}^{p/q},{ϒ}_{𝓁};\sigma \right)$, ${\hat{h}}_{p}$ is an n-dimensional vector with the l th element given by

(12) $${\left({\hat{h}}_{p}\right)}_{l}=\frac{1}{n_{p}}\sum_{i=1}^{n_{p}}K_{il}^{p}.$$

Similar to the work [44], the global optimal solution $\hat{\theta }$ can also be analytically obtained as

(13) $$\hat{\theta }={\left(\hat{H}+\lambda I_{n}\right)}^{-1}{\hat{h}}_{p},$$

where $I_{n}$ denotes an n-dimensional identity matrix.

Finally, we can apply Equation (13) to estimate the density ratio $r_{\alpha }(ϒ)$ using the estimator $\hat{r_{\alpha }}(ϒ;\theta )=\sum_{𝓁=1}^{n}{\hat{\theta }}_{𝓁}K\left(ϒ,{ϒ}_{𝓁}\right)$, and then calculate the Pearson-like scaled Bregman divergence ${\hat{D}}_{PL}\left(p\|q;m_{\alpha }\right)$ as defined in Equation (2.2).

Algorithm 1 outlines the change-point detection procedure using the Pearson-like scaled Bregman divergence (PLsBD) and kernel regression model: first, construct sliding windows of the data; second, identify the optimal values of the kernel width σ and regularization parameter λ using an $n_{f}$-fold cross validation method; third, estimate the parameters θ and density ratio $\hat{r_{\alpha }}(ϒ;\theta )$; finally, compute the Pearson-like scaled Bregman divergence; if the divergence score is beyond a given percentile threshold η, a change-point candidate is identified; move the sliding windows and repeat this procedure.

**Algorithm 1:** Pearson-like scaled Bregman divergence-based change-point detection

**Input:** Time series data; α: mixture value in measure $m_{\alpha };n:$ number of kernel functions; $n_{f}$: number of folds to use in the cross-validation; η: percentile threshold (0, 1)…
Step 1: Construct sliding windows
Divide time series data into consecutive segments ${ϒ}^{p}$, ${ϒ}^{q}$ with length $n_{p}$ and $n_{q}$.
Step 2: Identify optimal values of σ and λ
**For** each σ, λ value, apply $n_{f}$-fold cross-validation to
Calculate the kernel K(;σ);
Calculate $\hat{H}$ and ${\hat{h}}_{p}$ in Equation (11) and Equation (12);
Minimize the loss functions J(θ) in Equation (10) to identify optimal σ and λ.
Step 3: Estimation of density ratios
Calculate $\hat{\theta }$ and kernel K(;σ) using optimal σ and λ;
Calculate $\hat{r_{\alpha }}(ϒ;\theta )=\sum_{𝓁=1}^{n}{\hat{\theta }}_{𝓁}K\left(ϒ,{ϒ}_{𝓁}\right)$.
Step 4: Estimation of Pearson-like scaled Bregman divergence (PLsBD)
Estimate ${\hat{D}}_{PL}\left(p\|q;m_{\alpha }\right)\approx \frac{1}{2n_{p}}\sum_{i=1}^{n_{p}}\left({\hat{r}}_{\alpha }\left({ϒ}_{i}^{p}\right)\right)-\frac{2-\alpha }{2(1-\alpha )n_{q}}\sum_{i=1}^{n_{q}}\left({\hat{r}}_{\alpha }\left({ϒ}_{i}^{q}\right)\right)+\frac{1}{2(1-\alpha )}$.
Move the sliding windows forward to repeat the above steps until the end of data.
Step 5: Identify change points using PLsBD score
Sort the PLsBD score ${\hat{D}}_{PL}\left(p\|q;m_{\alpha }\right)$ and select the change-point candidates according to the percentile η
**Output:** Multiple change points.

Algorithm 1 is capable of computing the Pearson-like scaled Bregman divergence (PLsBD) score and detecting change points using the kernel regression model from multi-dimensional data. However, in multivariate time series data, not all covariates undergo transitions simultaneously. This phenomenon is especially pronounced in high-dimensional omics data, such as those encompassing thousands of genes but with only tens of time points. For instance, during cell cycle progression or disease evolution, only a subset of genes may exhibit notable changes in expression levels, while the majority remain relatively stable or undergo minor alterations.

Incorporating all covariates (e.g., thousands of genes) in change-point detection may result in biased outcomes, as a substantial number of minimally changing or non-changing covariates can influence the estimation of dissimilarity scores. To tackle this challenge, Algorithm 2 employs a random sampling strategy. In this approach, a subset of covariates is randomly sampled multiple times and used as input for Algorithm 1 to identify change-point candidates iteratively. The mean dissimilarity score across all iterations is then utilized for change-point detection, and the time points with the highest frequency are designated as the change-point candidates. This strategy effectively diminishes or alleviates the influence of numerous non-changing or minimally changing covariates on the obtained results, and identifies the time intervals for the change points.

**Algorithm 2:** High-dimensional PLsBD-based change-point detection with random sampling

**Input:** High-dimensional time series data with d covariates: $X=\left\{x_{1},x_{2},\ldots ,x_{d}\right\}$; number of random samples: $n_{\text{samples}}$;
**Repeat** $n_{\text{samples}}$ times
• Randomly sample a small subset of covariates from X;
• Apply Algorithm 1 to estimate the PLsBD dissimilarity score;
• Identify change-point candidates on each sampled subset.
**End Repeat**
Calculate the mean PLsBD dissimilarity score;
Identify candidates with the highest frequency.
**Output:** Multiple time intervals for change points

## 3. Results

For the results in this section, we employed R programming to implement the proposed method based on Pearson-like scaled Bregman divergence (PLsBD) and applied it to detect change points. We evaluated its performance on synthetic data and real-world microarray data obtained from the Drosophila life cycle. We assessed its performance by comparing it with the RuLSIF method, which has superior performance among existing density ratio estimation methods, uLSIF, and a well-known INSPECT algorithm for high-dimensional change-point detection.

For the data analysis, we constructed the kernel model using n=50 basis functions and utilized an $n_{f}=5$-fold cross-validation for all experiments to determine the optimal values of σ and λ. The parameter *step* defined the number of subsequences that preceded and followed a given time point, which were used to calculate the PLsBD score, and its value was typically set to around 5–10% of the length of the time series data. We set the percentile threshold value η to 0.9 by default, unless specified otherwise. This value represented the percentile above which a score was considered a potential change point. Furthermore, we tuned the values of α and window size to evaluate the parameter sensitivity in the change-point detection. The data were sampled using a sliding window that moved progressively from left to right, iteratively shifting along the time series data. At each iteration, the window advanced forward to encompass a new segment of the data. This process continued until the entire time series data were traversed, allowing for the comparison of density ratios between adjacent segments of data to identify potential change points.

### 3.1. Synthetic Data Analysis

#### Dataset 1: Time series Gaussian signal.

The synthetic time-series Gaussian signal consisted of four segments of data with an equal length of 100, and the data were generated from four different normal distributions: 𝒩(0,1),𝒩(10,1),𝒩(−5,1), and 𝒩(10,1), respectively. Thus, the changes in means occurred at time 101, 201, and 301, which were the predefined change points.

**Figure 1.**
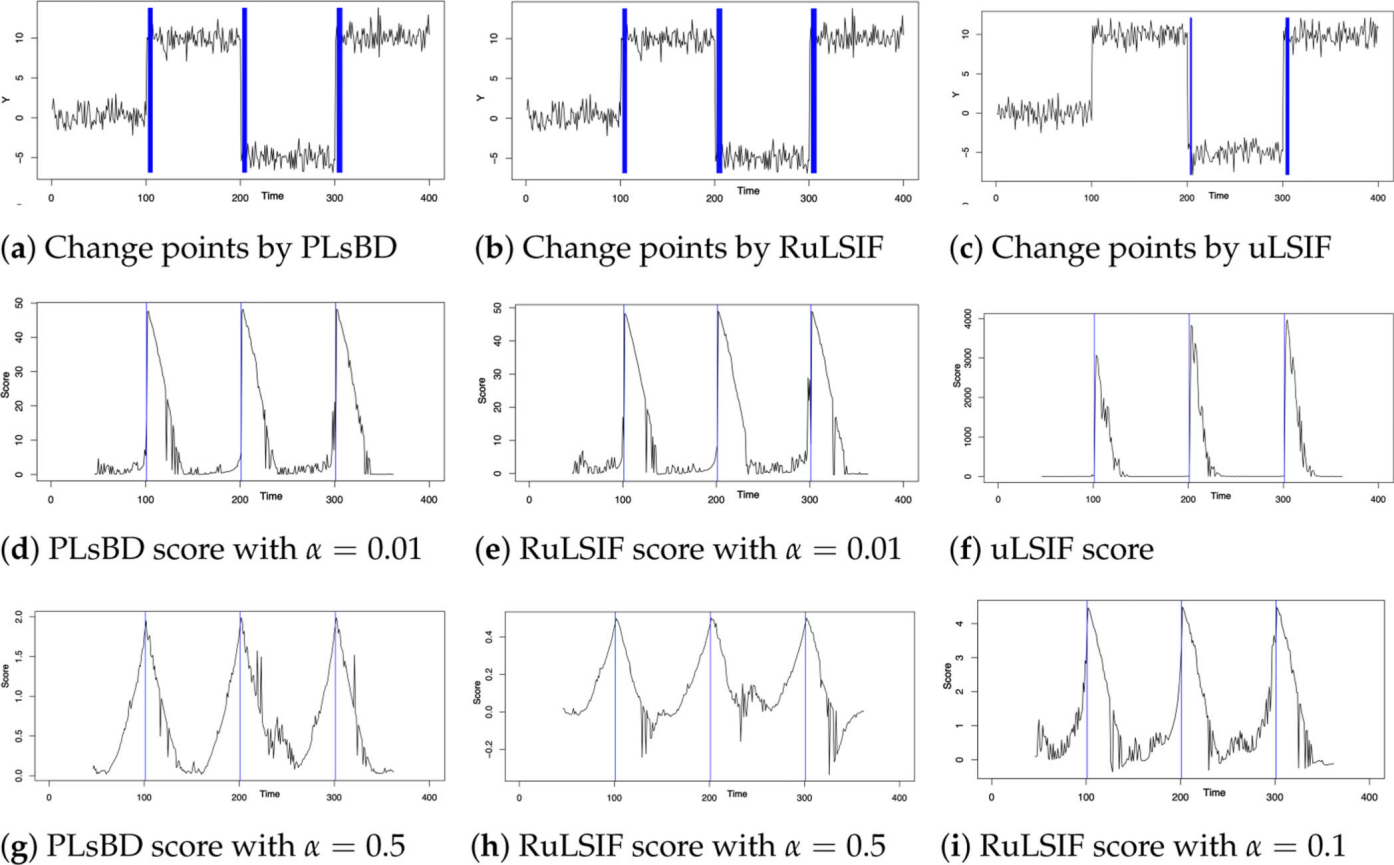
Gaussian signal’s change-point detection using the Pearson-like scaled Bregman divergence (PLsBD), RuLSIF, and uLSIF methods. (**a**–**c**) present the baseline results, and the blue color lines highlight the change-point candidates. (**d**–**f**) display the scores measured by the PLsBD (**d**), α-relative Pearson divergence with α=0.01 (**e**), and Pearson divergence (**f**). (**g**–**h**) show the dissimilarity scores using α=0.5 for both PLsBD and RuLSIF methods and α=0.1 for the RuLSIF method only (**i**).

Figure 1a–c display the baseline results, revealing that both PLsBD and RuLSIF methods accurately identified the change points. In contrast, the uLSIF method only identified two change points and missed the first one. Figure 1d–f depict the dissimilarity scores measured by the PLsBD (d), RuLSIF (e) using the value α=0.01, and uLSIF (f). In our baseline studies, we selected α=0.01 based on prior RuLSIF research [44,45], which recommended smaller α values. This choice was made because larger α values might induce numerical instabilities in the RuLSIF algorithm computations, potentially leading to negative divergence scores. The notably high divergence score in Figure 1f obtained using the uLSIF method (corresponding to α=0 in RuLSIF) can be attributed to the weakness of the Pearson divergence. This is because the density ratio function p/q in the Pearson divergence may become very large or unbounded under certain conditions. A change-point candidate is selected if its dissimilarity score exceeds a predefined percentile threshold η times the maximum dissimilarity score. However, the significantly large peak value around time t=201 in Figure 1f prevents the identification of the first peak around t=101 as a change-point candidate.

Figure 1d,e illustrate that for very small values of α, the dissimilarity scores calculated by both PLsBD and RuLSIF methods were highly similar, indicating comparable performance between these two methods. Theoretically, both PLsBD and RuLSIF scores describing the dissimilarity between the distributions should always be non-negative. However, in Figure 1g–i, it is evident that as α increased to 0.1 or greater, the RuLSIF score transitioned from positive to negative values at certain time points. That is, the RuLSIF method exhibited numerical instabilities when the value of α was large, underscoring its high sensitivity to α, a phenomenon also observed by other researchers. This numerical instability explains why a smaller α value is always chosen for the RuLSIF algorithm. On the contrary, the PLsBD score consistently maintained its non-negative property, even when α was around 0.5. This indicates that the PLsBD method is less sensitive to variations in α and has greater stability in numerical calculations compared to the RuLSIF method. In Figure 1g,h, we also observed that the PLsBD method yielded a relatively larger divergence value than the RuLSIF method when the same value of α was chosen. This observation is consistent with the theoretical findings of Lemma 3, which suggest that the α-relative Pearson divergence serves as a lower bound for the PLsBD method.

**Figure 2.**
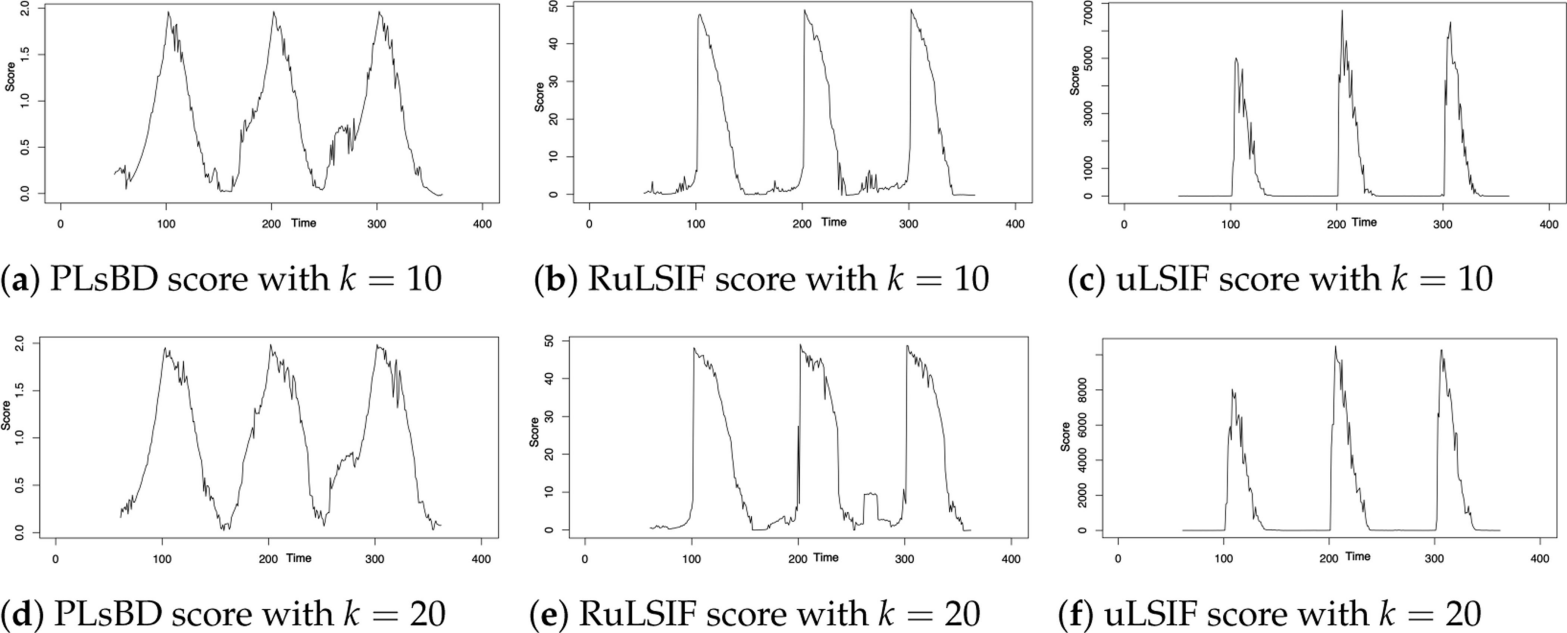
Dissimilarity scores were computed with varying window size k=10,20 using the PLsBD (**a**,**d**), RuLSIF (**b**,**e**), and uLSIF (**c**,**f**) methods.

The data were sampled using a sliding window progressing from left to right by iteratively shifting the window along the time series data. At each iteration, the window was moved one step forward, covering a new segment of the time series. This process continued until the entire time series was traversed, allowing for the comparison of density ratios between adjacent windows to identify potential change points. The window size, denoted by k, is an important parameter that might influence the accuracy of change-point detection in different density ratio estimation methods. Figure 1 uses a smaller window size value of k=5. Next, we increased k to 10 and 20, and Figure 2 depicts the dissimilarity score versus the change in window size k. These figures indicate that a smaller window size produces sharper peaks in dissimilarity scores, potentially aiding in the detection of subtle changes in the data. Conversely, a larger window size yields smoother or flatter peaks, indicating a larger change-point interval, which may increase the likelihood of identifying more change-point candidates. From our simulation studies, we recommend selecting the optimal window size within the range of 5 to 10 for robust and accurate change-point detection. In our subsequent microarray data analysis, we chose eight as the optimal window size.

#### Dataset 2: auto-regressive model.

The one-dimensional auto-regressive model AR(2), similar to [44], was used to generate 1000 samples (t=1,…,1000):

$${ϒ}_{t}=0.6{ϒ}_{t-1}-0.5{ϒ}_{t-2}+\epsilon_{t}$$

where $\epsilon_{t}\sim 𝒩(0,1)$. The initial values were set to ${ϒ}_{1}={ϒ}_{2}=0$. A change point was inserted at every 100 time steps by increasing the noise mean μ by two, that is, $\mu_{t}=\mu_{t-100}+2$, where t=100 N+1, where N=1,…,9, that is, there were nine change points.

**Figure 3.**
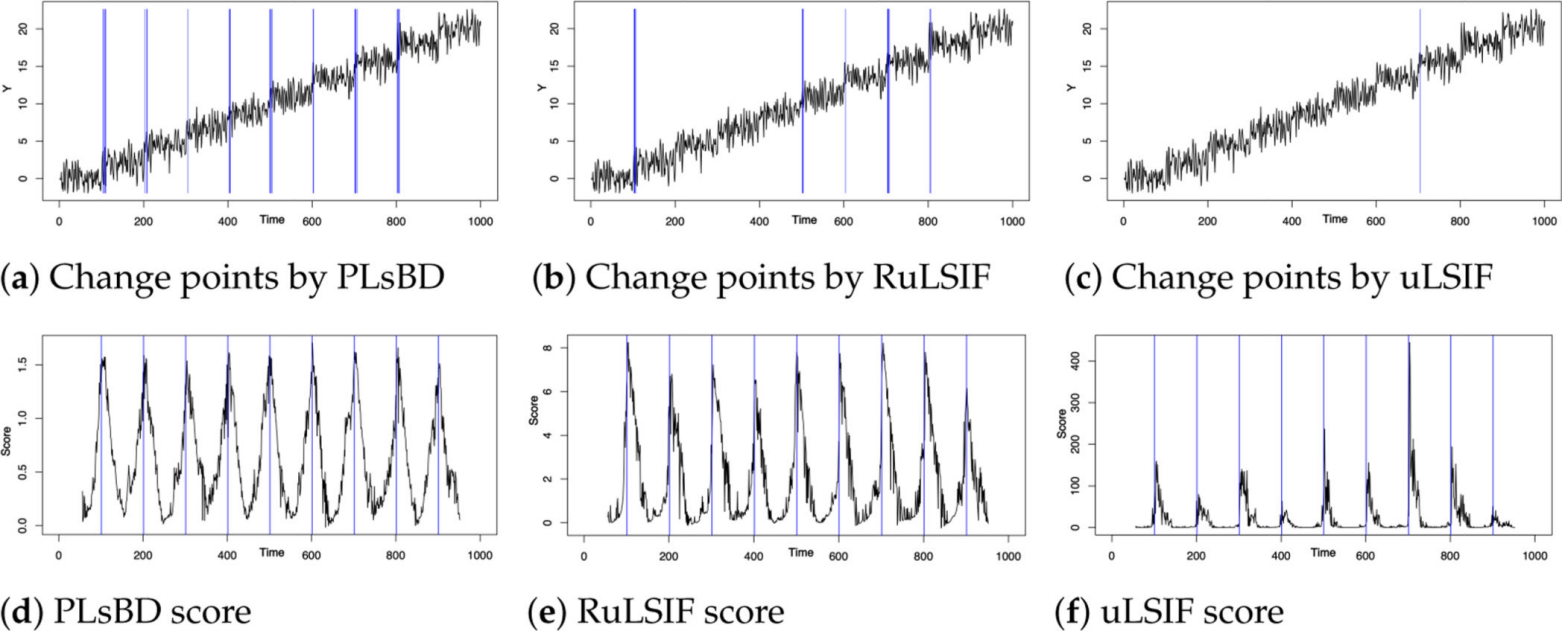
Change-point detection within time series data generated by the auto-regressive model AR(2). Panels (**a**–**c**) visualize the time series data with identified change-point candidates highlighted by blue rectangles. Panels (**d**–**f**) display the dissimilarity scores using the PLsBD (**d**), RuLSIF (**e**), and uLSIF (**f**) methods. The true change points are marked by blue lines.

Figure 3a–c visualize the time series data generated by the aforementioned autoregressive model, featuring nine change points. The identified change-point candidates are highlighted by blue rectangles using the PLsBD (a), RuLSIF (b), and uLSIF (c) methods with parameters η=0.9, *step* = 50, and window size k=5. In the PLsBD baseline simulation, we intentionally set α=0.5 to assess the robustness of the PLsBD to this parameter. Additionally, Lemma 2 showed that PLsBD was a symmetric measure when α=0.5, which is advantageous for change-point detection algorithms. However, to prevent the occurrence of negative RuLSIF scores, we selected a smaller α value for the RuLSIF algorithm, specifically, α=0.5 in this baseline study. Figure 3a–c show that in the baseline results, PLsBD correctly identified eight change points, whereas RuLSIF and uLSIF only detected five and one change point, respectively. Figure 3d–f illustrate the dissimilarity scores obtained from the PLsBD, RuLSIF, and uLSIF methods, with the true change points marked by blue lines. All these methods showed nine peaks of dissimilarity scores around the real change points. However, the magnitudes of all dissimilarity score peaks in the PLsBD were similar, while those in the RuLSIF and uLSIF methods differed. When we defined a percentile threshold η, only those peaks with a similar magnitude could potentially be identified as change points by the algorithm. Typically, a smaller α value results in significantly higher divergence scores, potentially hindering the identification of small peaks corresponding to certain change-point candidates.

The analysis of synthetic data for change-point detection demonstrates the PLsBD method’s superior performance and excellent numerical stability in computation compared to traditional density ratio methods such as RuLSIF and uLSIF. Additionally, when applied to multivariate time series synthetic data, the PLsBD also outperformed other methods (results not shown due to length). However, multiple studies [14,28,48,49] have indicated that change-point detection in high-dimensional real-world noisy data is particularly challenging, especially in genomics data with thousands of genes, limited time points, and substantial noise. Our next step was to apply Algorithm 2, which incorporates a random sampling strategy with Algorithm 1, to analyze high-dimensional microarray data from Drosophila and evaluate its performance against other CPD methods.

### 3.2. High-Dimensional Microarray Data Analysis

Arbeitman et al.’s study [50] measured the gene expression levels of 4028 genes of wild-type Drosophila across 67 successive time points throughout its life cycle: 31, 10, 18, and 8-time points for the embryo, larva, pupa, and adult stages, respectively. Thus, the four major stages of morphogenesis corresponded to the change points around the time points 32, 42 and 60. The study [50] also indicated an important point around 19–20, which represented an important morphological transition during mid-embryogenesis. This dataset has been widely used as a benchmark for evaluating various change-point detection algorithms in numerous studies [28,48]. However, due to the inherent noise in microarray data and the fact that the number of genes (4028) far exceeds the number of observations (67), no algorithm can precisely identify all these change points at present. Therefore, most studies opt to use a smaller subset of data consisting of eleven marker genes involved in the wing muscle development, as identified by Zhao and Dondelinger et al.’s work [51,52], to detect change points. Next, we first employed our PLsBD approach to identify change points in the time series microarray data of these 11 genes.

#### Dataset 3: microarray data of 11 genes involved in Drosophila’s wing muscle development.

Various methods [28,48,49] have been developed to study the time-varying genetic networks regulating the wing muscle development in the life cycle of Drosophila. Therefore, change-point detection is the critical initial step preceding network reconstruction. Despite the significant reduction in the number of genes, change-point detection remains challenging. This is because the expression levels of these genes may not change synchronously, and there are very few observations in the stages of larva and adult. For instance, the TESLA algorithm [53] and non-stationary dynamic Bayesian network method [48] failed to detect the change point of the transition from pupa to adult, while the ARTIVA and INSPECT methods [27,28,49] missed the change point of the transition from larva to pupa.

In this work, each time, we randomly selected 3–5 genes from the pool of 11 available genes for 300 iterations while fixing the windows size k=8 and *step* = 5, and η=0.9. Subsequently, we applied Algorithm 2 to compute the mean PLsBD scores and the frequencies of all potential change-point candidates with varying values of α from 0.01 to 0.5. Our goal was to identify the transition points at 20, 32, 42, and 60, corresponding to the stages of mid-embryogenesis, embryo–larva, larva–pupa, and pupa–adult. Additionally, we employed the RuLSIF method for comparison purposes, using smaller α values to prevent the occurrence of a negative RuLSIF divergence.

**Figure 4.**
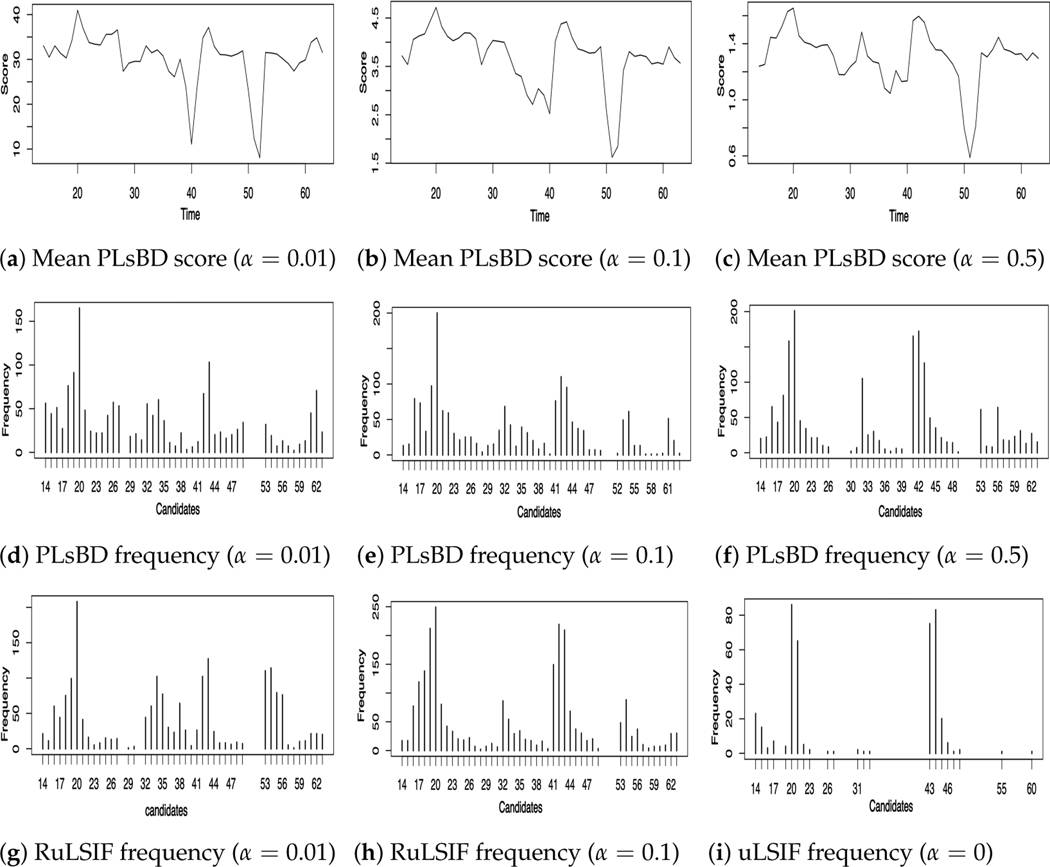
Change points inferred from time series microarray data of eleven genes involved in Drosophila’s muscle development. Panels (**a**–**c**) plot the mean dissimilarity score of PLsBD with different values of α. Panels (**d**–**f**) and (**g**–**i**) plot the frequencies of each change-point candidate using the PLsBD and RuLSIF methods.

Figure 4 illustrates the mean dissimilarity scores (a–c) obtained using the PLsBD algorithm and the frequency distribution of change-point candidates using random sampling for both PLsBD (d–f) and RuLSIF (g–i) methods. The mean PLsBD scores depicted in Figure 4a–c reveal that the peaks occurred around the true change points, indicating potential change-point candidates located at the intervals 19–20, 31–33, 41–43, 55–56, and 61–62. These figures demonstrate that the PLsBD score remained robust across different values of α, as they exhibit similarity across all three values. Furthermore, the frequency distribution shown in Figure 4d–f confirms that these intervals had relatively highest frequencies, indicating that they likely contained the true change points when using the PLsBD-based random sampling method. For comparison, we incorporated the RuLSIF method with the random sampling approach and performed a similar procedure using a smaller α value to prevent negative divergence scores. Figure 4g–i demonstrate that when α=0, corresponding to the uLSIF method, it only identified two change-point intervals from the frequency distribution. The majority of identified change-point candidates clustered around 20–21 and 43–44. When α ranged from 0.01 to 0.1, the RuLSIF method successfully identified most of the change-point intervals, exhibiting comparable performance to the PLsBD in identifying most of the change points. The simulation studies also suggested that both the number of iterations and sampled genes significantly impacted the frequency distribution of the change points, particularly influencing the height of the peak around 60. Our method identified change-point intervals almost similar to Lebre et al.’s work [49] which assessed the performance of ARTIVA and TESLA algorithms on the 11-gene data, indicating that our approach is as competitive in detecting change points within the 11-gene dataset as existing approaches. Our mean PLsBD score results in Figure 4a–c also suggested a possible change point around 53–54, which was similar to the ARTIVA and Bayesian methods’ results [49,54]. This change point might indicate a late-pupa stage, which is partially explained by a recent study [55] that the late pupae are more similar to adults of Drosophila.

#### Dataset 4: microarray data comprising 4028 genes of the Drosophila life cycle.

Detecting change points from high-dimensional genomics data poses a considerable challenge, particularly when the number of genes far exceeds the available time points and when there is significant noise present. A preliminary analysis indicated that only a subset of genes displayed noticeable alterations at different stages of the cell cycle, and most genes did not change synchronously, which can influence the estimation of dissimilarity scores. To address this challenge, most existing approaches either manually select a specific set of genes (e.g., Dataset 3) or apply dimension reduction techniques (e.g., clustering or co-expressed network analysis) to mitigate the complexity introduced by the vast number of genes.

**Figure 5.**
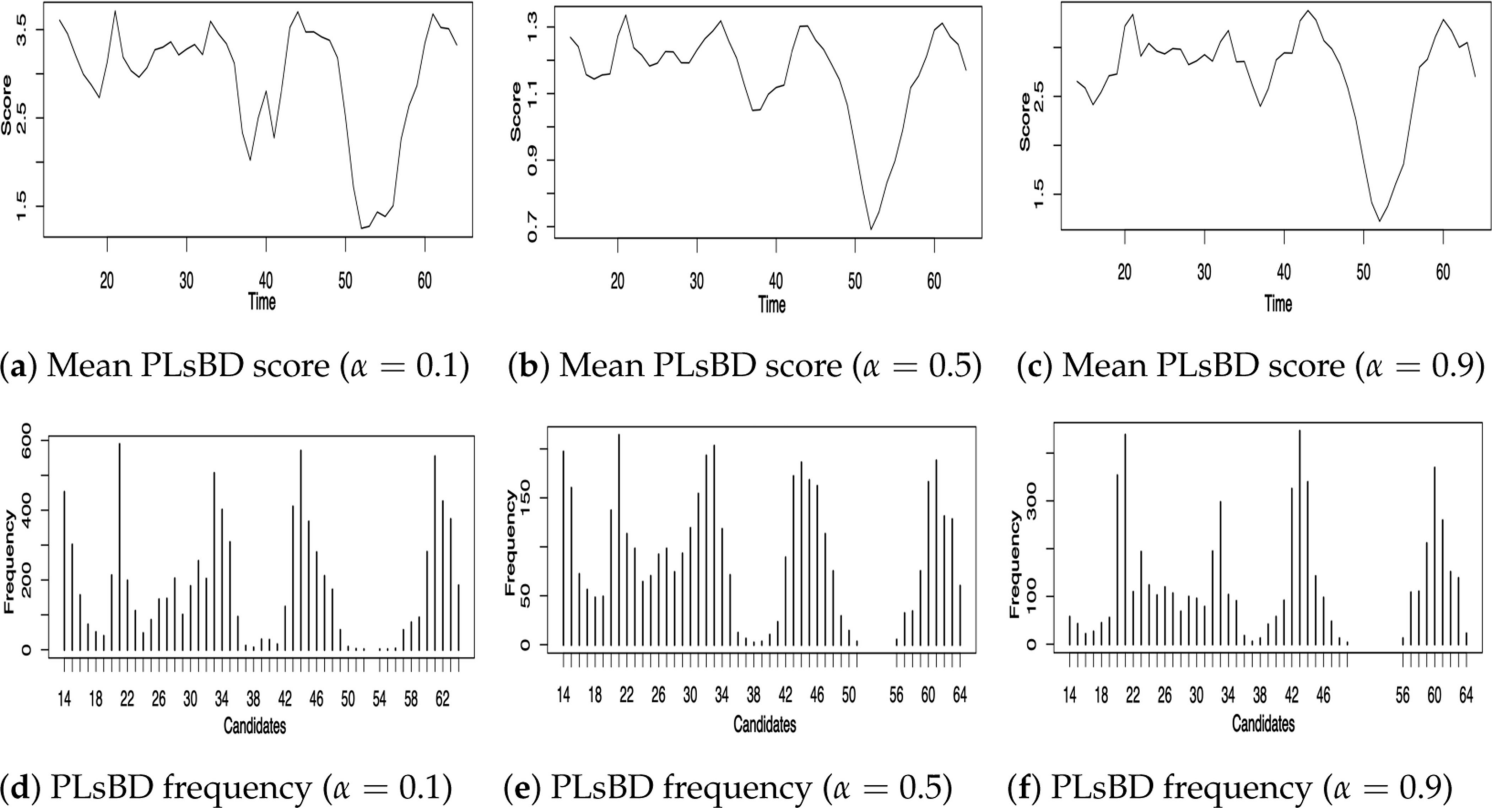
Change points inferred from time series microarray data of 4028 genes in Drosophila’s life cycle. Panels (**a**–**c**) plot the mean PLsBD dissimilarity score (**a**–**c**) with different values of α, calculated by randomly sampling 40 genes 1000 times. Panels (**d**–**f**) plot the frequencies of each change-point candidate.

In this study, we repetitively and randomly selected 40 genes out of the 4028 genes for 1000 iterations, maintaining fixed parameter values as in the analysis of Dataset 3. We then applied Algorithm 2 to identify the change-point intervals. Figure 5 illustrates the mean PLsBD dissimilarity scores (a–c) and the frequency distribution (d–f) of all potential change-point candidates, varying the value of α from 0.1 to 0.9 to assess its robustness. The mean PLsBD dissimilarity score plot in Figure 5a–c demonstrates four distinct peaks, indicating the presence of four change points in the data. Moreover, the PLsBD score maintained its non-negative property even when α was set to 0.9, highlighting its robustness to variations in α, which gives the PLsBD an advantage in choosing a wide range of α values over the RuLSIF method. Additionally, the frequency plots (d–f) identified four time intervals, 20–22, 32–34, 42–44, and 60–62, with the highest frequencies encompassing the true change points. Despite the larger dataset comprising 4028 genes, the performance remained comparable to the results obtained from the analysis of 11 genes, suggesting the robustness of the PLsBD method in detecting change points across different scales of data.

**Figure 6.**
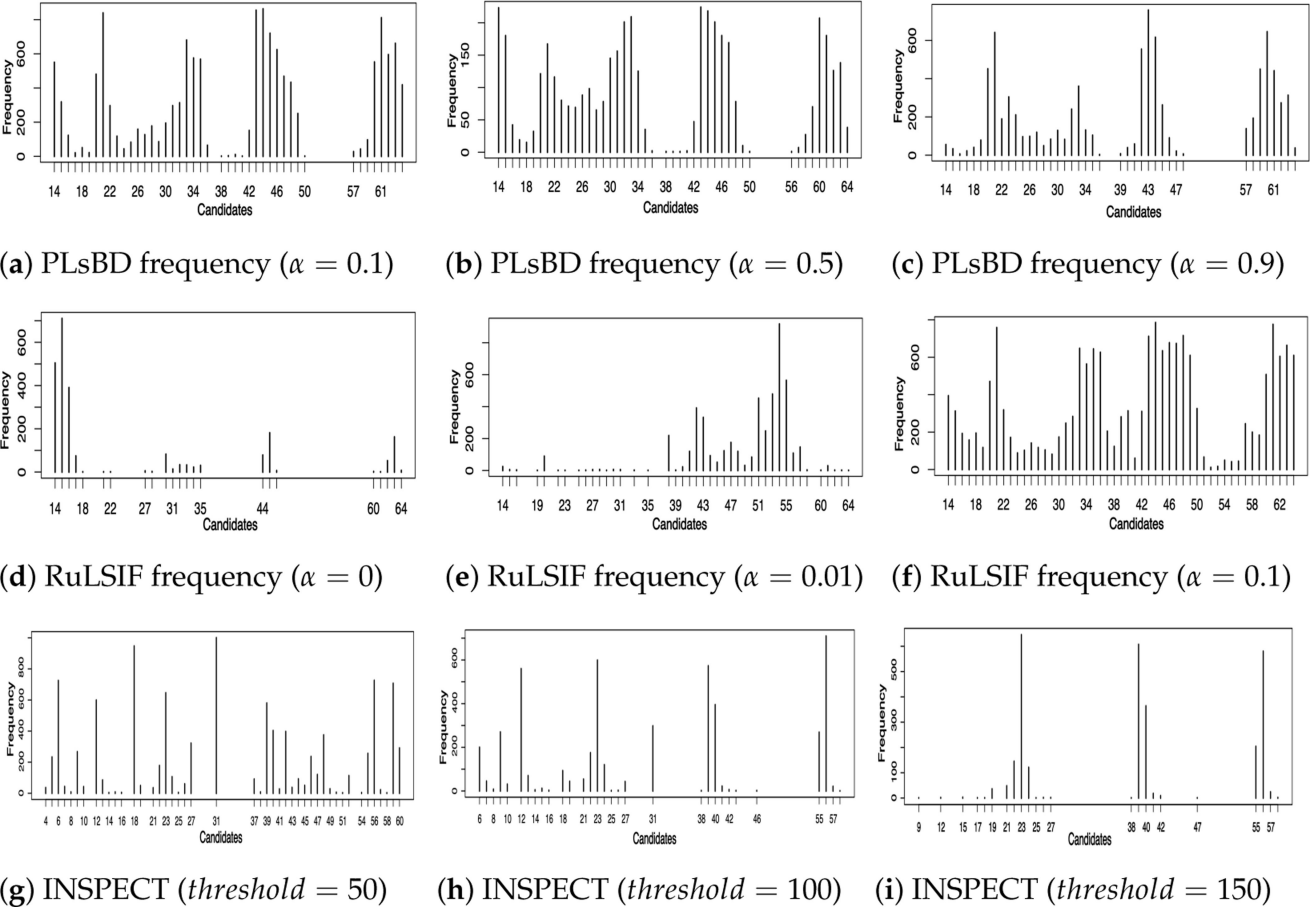
Frequency distribution of change-point candidates using 400 genes randomly sampled 1000 times from a pool of 4028 genes using the PLsBD (**a**–**c**), uLSIF (**d**), RuLSIF (**e**–**f**), and INSPECT (**g**–**i**) methods. Parameters were fixed at *ste* p=5, η=0.9, and k=8 for the PLsBD, uLSIF, and RuLSIF methods. Panels (**g**–**i**) display the frequencies of each change-point candidate using the INSPECT method with different values of *threshold*.

To further assess the scalability of the PLsBD method, we randomly sampled 400 genes from a pool of 4028 genes at each iteration and applied various methods, including the PLsBD, uLSIF, RuLSIF, and INSPECT methods, to make the testing more challenging and detect change points. This process was repeated 1000 times to ensure the robustness and reliability of the results. The parameter values for PLsBD, uLSIF, and RuLSIF methods were kept consistent with the analysis performed in Dataset 3. Figure 6 illustrates the frequency distribution of change-point candidates using the PLsBD (a–c), uLSIF (d), RuLSIF (e–f), and INSPECT (g–i) methods. Our studies persistently demonstrated that even with a significant increase in the dimensionality of the data, PLsBD could still successfully identify most of the time intervals of change points across a range of α values from 0.1 to 0.9 in Figure 6a–c. However, in the RuLSIF studies, baseline results (now shown) revealed that the divergence score could become negative if α was large. When varying the values of α from 0 to 0.1, the results in Figure 6d–f exhibited poorer performance compared to the PLsBD results in Figure 6a–c. The change-point candidates identified using the uLSIF method illustrated a significant concentration of candidates around the time interval of 14–16, capturing over 80% of the total candidates. In contrast, a minimal number of candidates were close to the true change time points around 42 and 60. This indicates the uLSIF method’s limitation to accurately identify change points in the high-dimensional data. This discrepancy partially arises from the unboundedness issues inherent in the Pearson divergence utilized in the uLSIF method, which can lead to inaccuracies or inconsistencies in detecting change points, particularly in high-dimensional datasets. Figure 6g–i also show the performance of the INSPECT algorithm, a widely used high-dimensional change-point estimation method based on the CUSUM technique. The INSPECT method’s performance is contingent upon the selection of a crucial parameter known as the *threshold*. A lower *threshold* value (Figure 6g) prompts the INSPECT method to identify numerous change-point candidates, including some true change points. On the contrary, as the *threshold* value increases (Figure 6i), the INSPECT method may overlook certain change points. In contrast to the INSPECT algorithm, our PLsBD approach, coupled with random sampling, exhibits a higher degree of robustness and small sensitivity to parameter adjustments, which ensures the detection of change points with greater accuracy, even in high-dimensional datasets.

## 4. Discussion

In this work, we developed a novel Pearson-like scaled Bregman divergence (PLsBD) coupled with a random sampling method for change-point detection in high-dimensional time series data. Our PLsBD method can overcome several limitations inherent in traditional density estimation-based change-point detection methods, including KLIEP, uLSIF, and RuLSIF, which rely on the Kullback–Leibler (KL) divergence, Pearson divergence, and α-relative Pearson divergence, respectively. Our mathematical studies proved that the Pearson-like scaled Bregman divergence satisfied metric properties under certain conditions. After introducing a mixture measure, we derived an analytical result expressed as a function of the relative density ratio to estimate the dissimilarity between individual distributions, which can be estimated using Kernel regression method. Finally, we integrated the PLsBD algorithm with a random sampling approach to analyze synthetic and high-dimensional microarray data. Our PLsBD method demonstrated enhanced sensitivity to temporal changes and excellent numerical stability in computation, and it could accurately identify most change-point intervals in high-dimensional datasets, such as those with 4028 genes. In comparative analyses, it consistently outperformed traditional methods like uLSIF, RuLSIF, and INSPECT, particularly when applied to datasets with numerous covariates and limited time points. This superior performance underscores the potential of PLsBD as a robust tool for change-point detection in diverse data settings. The Pearson-like scaled Bregman divergence (PLsBD) can also be applied for anomaly detection in various domains, given its capability to estimate the dissimilarities between distributions. Additionally, integrating the PLsBD with some network inference algorithms, for example, dynamic Bayesian network, could facilitate time-varying network inference in evolving systems. In real-world applications, detecting change points becomes particularly challenging when dealing with high-dimensional datasets containing a substantial number of missing values. Recently, the integration of imputation methods with CPD algorithms has been explored for change-point detection [56], However, this approach has its limitations due to the assumption that the data are modeled as a Gaussian copula [57]. The proposed PLsBD method is limited to retrospective change-point detection and does not support online datasets with missing values. Our future research will focus on extending the PLsBD approach and integrating it with some imputation methods, such as fGAIN [58], MAGIC [59], and SAVER [60], for online change-point detection in high-dimensional datasets with missing values.

## Data Availability

The R code and data for the proposed method are available on GitHub: https://github.com/TongSi98/PLsBD-CPD (accessed on 8 May 2024).

## Abbreviations

CPD: Change-point detection
PLsBD: Pearson like scaled Bregman divergence
RuLSIF: Relative unconstrained least-squares importance fitting
INSPECT: Informative sparse projection for estimation of change points
